# Supplementary material for: Population Study of Ovarian Cancer Risk Prediction for Targeted Screening and Prevention
Source: Cancers (Basel). 2020 May 15;12(5):1241. doi: 10.3390/cancers12051241 (PMC7281662; doi:10.3390/cancers12051241)
Supplement: Supplementary file 1 [file cancers-12-01241-s001.pdf]

cancers

Population Study of Ovarian Cancer Risk Prediction for Targeted Screening and Prevention

Faiza Gaba, Oleg Blyuss, Xinting Liu, Shivam Goyal, Nishant Lahoti, Dhivya Chandrasekaran, Margarida Kurzer, Jatinderpal Kalsi, Saskia Sanderson, Anne Lanceley, Munaza Ahmed, Lucy Side, Aleksandra Gentry-Maharaj, Yvonne Wallis, Andrew Wallace, Jo Waller, Craig Luccarini, Xin Yang, Joe Dennis, Alison Dunning, Andrew Lee, Antonis C. Antoniou, Rosa Legood, Usha Menon, Ian Jacobs and Ranjit Manchanda

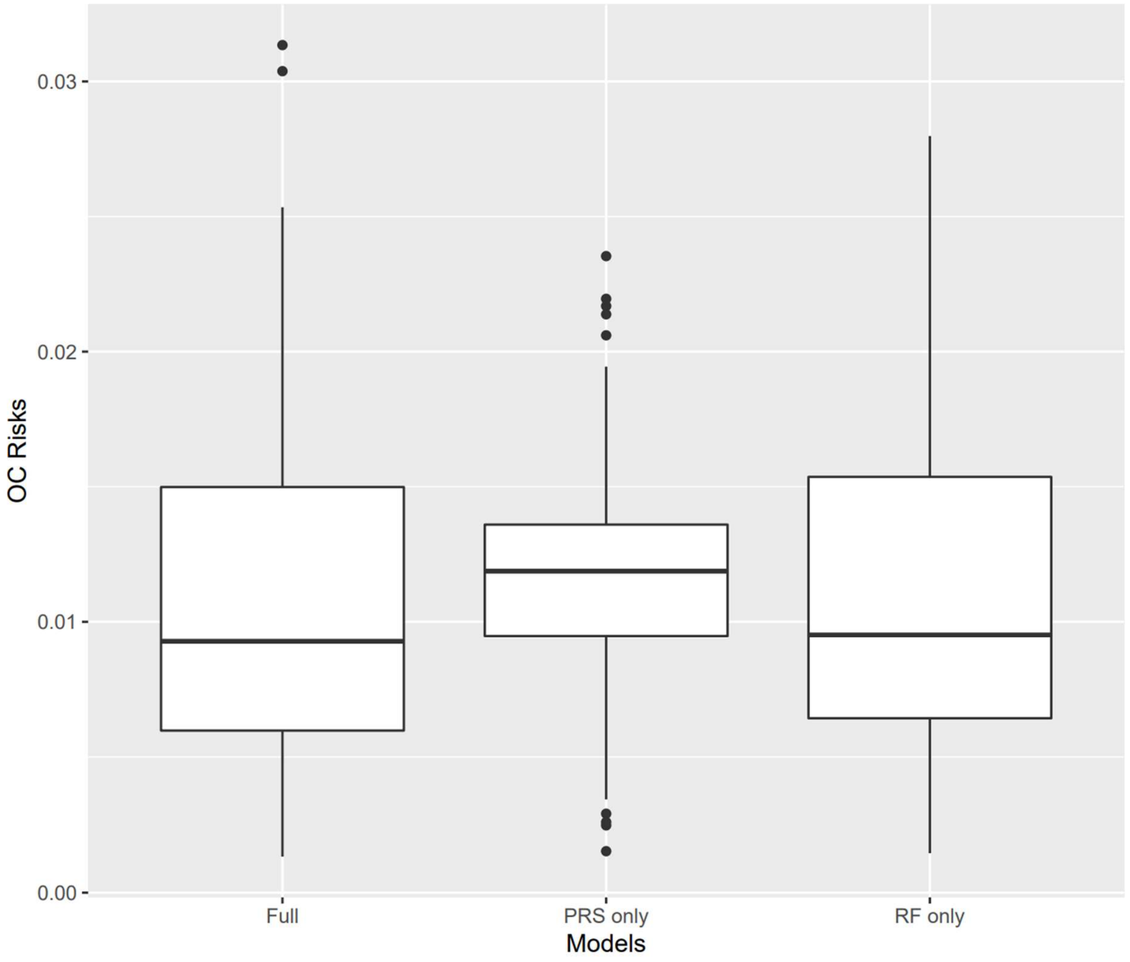

Figure S1. Distributions of the remaining lifetime ovarian cancer risks given by different model versions. Model predictions after excluding the identified *BRCA1* mutation carrier and always considering family history information and that women do not carry a rare pathogenic ovarian cancer susceptibility variant. Models considered: Full – predictions under the full model; Polygenic Risk Scores (PRS) only – predictions considering the PRS only; Risk Factors (RF)- predictions considering only the epidemiological risk factors.

Table S1. Perceived benefits, risks, limitations or panel genetic testing/ovarian cancer risk assessment measured at baseline.

	Not at all important		Somewhat important		Very important		Does not apply	
**Benefits**	%	N	%	N	%	N	%	N
To be reassured	4.9	5/102	46.08	47/102	48.04	49/102	0.98	1/102
To enhance cancer prevention	1.96	2/102	16.67	17/102	80.39	82/102	0.98	1/102
To learn about my children's risk.	3.92	4/102	15.69	16/102	54.95	56/102	25.49	26/102
To make a decision about surgery to have my ovaries and tubes removed before cancer arises.	13.27	13/98	32.65	32/98	44.90	44/98	9.18	9/98
To benefit research or improve the health of other women	1.01	1/99	17.17	17/99	80.81	80/99	1.01	1/99
To make childbearing decisions	20.62	20/97	10.31	10/97	14.43	14/97	54.64	53/97
To know if I need to get cancer screening tests more often.	8	8/100	28	28/100	61	61/100	3	3/100
To reduce uncertainty	11.11	11/99	38.38	38/99	49.49	49/99	1.01	1/99
**Risks/limitations**								
I am worried about losing my insurance.	49	49/100	19	19/100	9	9/100	23	23/100
I am worried about loss of confidentiality.	67.33	68/101	18.81	19/101	5.94	6/101	7.92	8/101
I am worried about stigmatization.	85	85/100	7	7/100	2	2/100	6	6/100
I do not trust modern medicine.	74.75	74/99	5.05	5/99	2.02	2/99	18.18	18/99
I believe that there is nothing that can be done to prevent getting cancer.	62.63	62/99	9.09	9/99	6.06	6/99	22.22	22/99
I am concerned about the effect it would have on my family.	34.65	35/101	40.59	41/101	15.84	16/101	8.91	9/101
I am concerned that I could not handle it emotionally.	50.50	51/101	30.69	31/101	6.93	7/101	11.88	12/101
I am concerned that genetic information from the test might be used to single out individuals of a particular ethnic group	76.77	76/99	9.09	9/99	2.02	2/99	12.12	12/99

Table S2. Decision aid question responses for women who consented and who did not consent to the study.

Decision aid questions	Responses to individual questions												*p*-value (Fisher's exact test)
	Individuals who consented						Individuals who did not consent						
	Against taking part		In favour of taking part		Neither in favour nor against taking part		Against taking part		In favour of taking part		Neither in favour nor against taking part		
	%	N	%	N	%	N	%	N	%	N	%	N	
My current worries about ovarian cancer make me feel...	0	0/103	83.5	86/103	16.5	17/103	0	0/20	75	15/20	25	5/20	0.353
Having genetic testing as part of this programme makes me feel.	0	0/103	93.2	96/103	6.8	7/103	0	0/20	95	19/20	5	1/20	1

The possibility of learning about my risk of ovarian cancer as part of this study makes me feel…	0.97	1/103	95.15	98/103	3.88	4/103	0	0/20	95	19/20	5	1/20	1
Although most women will be reassured that they are at low risk for ovarian cancer as part of participating in this programme, some women may learn that they are at high risk. This makes me feel.	1.94	2/103	88.35	91/103	9.71	10/103	0	0/20	80	16/20	20	4/20	0.472
The possibility of learning genetic information that may be important for my family members makes me feel.	0	0/103	94.17	97/103	5.83	6/103	0	0/20	95	19/20	5	1/20	1
The benefits of taking part in this programme make me feel.	0	0/103	98.06	101/103	1.94	2/103	0	0/20	95	19/20	5	1/20	0.38
The risks of taking part in this programme make me feel.	3.88	4/103	64.08	66/103	32.04	33/103	0	0/20	60	12/20	40	8/20	0.81
My fears/worries/concerns about screening make me feel.	0.97	1/103	67.96	70/103	31.07	32/103	5	1/20	65	13/20	30	6/20	0.432
My fears/worries/concerns about the possibility of having surgery to remove my ovaries make me feel.	9.71	10/103	63.11	65/103	27.18	28/103	20	4/20	55	11/20	25	5/20	0.388
If I decide not to take part, I may regret my decision if I develop ovarian cancer in the future	**Disagree**		**Agree**		**Unsure**		**Disagree**		**Agree**		**Unsure**		
	**%**	**N**	**%**	**N**	**%**	**N**	**%**	**N**	**%**	**N**	**%**	**N**	
	9.71	10/103	88.35	91/103	1.94	2/103	10	2/20	75	15/20	15	3/20	0.036
**Mean of total scores** *	8.1						7.4						0.14 **

23/123 individuals viewed the decision aid more than once. For these 23 cases, the responses to the first view are included in this table. * The responses to the individual decision aid questions were scored accordingly: 1 = in favour of taking part, −1 = against taking part, 0 = neither in favour or against taking part, 1 = agree, −1 = disagree, 0 = unsure. Women with total scores between −10 and −1 were considered to be "leaning-against taking part"; scores between 0 and 5 "undecided"; and scores between 6 and 10 "leaning-towards taking part". ** Wilcoxon rank-sum test.

Table S3. Mean scores at repeat viewings for participants who viewed the decision aid on multiple occasions.

DA viewing attempt	Number of participants viewing DA on multiple occasions according to number of attempts (N)	Mean score (SD, range) at each attempt
**1st attempt**	23	7.739 (SD = 2.378, range 3–10)
**2nd attempt**	23	8.348 (SD = 2.058, range 2–10)
**3rd attempt**	17	8.588 (SD = 1.906, range 4–10)
**4th attempt**	13	7.846 (SD = 1.819, range 4–10)
**5th attempt**	6	8 (SD = 2.449, range 4–10)
**6th attempt**	3	9 (SD = 1.732, range 7–10)
**7th attempt**	1	10

DA – decision aid; SD – standard deviation. 23/123 individuals viewed the decision aid on more than one occasion. This table depicts the mean scores of these 23 individuals at baseline (first attempt) and at repeat viewing attempts.

Table S4. Linear random-effects mixed models for trends in decision aid scores in participants viewing the decision aid on multiple occasions.

Mixed models for trends	Coef.	Std. Err	$p > \lvert z \rvert$	95% CI
**BL attempt vs. attempt 2**	0.609	0.273	0.03	0.054 to 1.111
**BL attempt vs. attempt 3**	0.68	0.305	0.03	−0.002 to 1.294
**BL attempt vs. attempt 4**	0.356	0	0.295	−0.331 to 0.939
**BL attempt vs. attempt 5**	0.715	0.456	0.123	−0.183 to 1.599
**BL attempt vs. attempt 6**	0.701	0.615	0.259	−0.535 to 1.936
**BL attempt vs. attempt 7**	0.584	1.019	0.569	−1.308 to 2.655

BL – baseline; Coef. – coefficient; Std. Err – standard error; Univariate analysis comparing mean decision aid scores at baseline (first attempt) to repeat attempts for the 23/123 individuals who viewed the decision aid on multiple occasions.
